# Vaginal Microbiome Signature Is Associated With Spontaneous Preterm Delivery

**DOI:** 10.3389/fmed.2019.00201

**Published:** 2019-09-10

**Authors:** Keli Hočevar, Aleš Maver, Marijana Vidmar Šimic, Alenka Hodžić, Alexander Haslberger, Tanja Premru Seršen, Borut Peterlin

**Affiliations:** ^1^Clinical Institute of Medical Genetics, University Medical Centre Ljubljana, Ljubljana, Slovenia; ^2^Division of Obstetrics and Gynecology, Department of Perinatology, University Medical Centre Ljubljana, Ljubljana, Slovenia; ^3^Department of Nutritional Sciences, University of Vienna, Vienna, Austria; ^4^Medical Faculty, University of Ljubljana, Ljubljana, Slovenia

**Keywords:** preterm delivery, microbiome, next-generation sequencing, 16S rRNA gene, vaginal microbiome, pregnancy

## Abstract

**Background:** Preterm delivery (PTD) represents an important public health and therapeutic challenge. Despite the reported link between the composition of vaginal microbiome and PTD, previous studies were inconsistent in their conclusions and utilized non-uniform designs. We performed an independent case-control study carried out on the Slovenian population, where we re-evaluated the role of the vaginal microbiome in PTD.

**Methods:** Vaginal microbiomes of pregnant women who delivered preterm were compared to those delivered at term to examine differences in the microbial richness, diversity, and differential abundance of specific taxa. We obtained vaginal swab samples from 155 Caucasian women who were classified as either term (≥38^0/7^ weeks, *n* = 107) or preterm (≤36^6/7^ weeks, *n* = 48) in exclusion of any other medical or obstetric conditions. The vaginal microbiomes of these women were characterized by 16S ribosomal RNA (rRNA) gene sequencing of the V3-V4 region on the MiSeq platform.

**Results:** Women who experienced PTD had a higher microbial richness (Chao1, *P* = 0.011) and alpha diversity (Shannon, *P* = 0.00059) than women with term deliveries. We report that overall vaginal microbial community composition (beta-diversity) was significantly different by delivery gestational age category (*P*_WeightedUnifrac_ < 0.001). Women who delivered preterm had decreased *Lactobacilli* spp. abundance as well as increased abundance of *Gardnerella* and other bacterial vaginosis (BV) and aerobic vaginitis (AV) associated genera including *Atopobium, Sneathia, Gemella, Megasphaera, Dorea, Streptococcus*, and *Escherichia/Shigella*.

**Conclusions:** In the present study, we provide further evidence that vaginal microbiome composition is associated with PTD.

## Introduction

Preterm delivery (PTD), defined by the World Health Organization (WHO) as the delivery of an infant before completed 37 weeks of gestation (1), represents a major global public health burden. It is the leading determinant of neonatal morbidity and mortality in developed countries (2) and the second most common cause of death in children under the age of five worldwide (3). Annually, 5–18% of pregnancies end preterm, resulting in 15 million premature babies (3), and prevalence is still escalating. Moreover, survivor premature babies have an increased risk of neonatal complications associated with the immaturity of organ systems, such as respiratory distress syndrome, cerebral palsy, retinopathy, hearing impairment, and bronchopulmonary dysplasia as well as increased long-term risk for a spectrum of cognitive and neurologic disorders (4–6).

Underlying causes of PTD are not completely understood, with more than 65–75% spontaneous PTDs with idiopathic onset (2, 7). Thus, it is still challenging to identify women at higher risk. Epidemiologic data suggest that complex genetic, environmental and sociodemographic factors are involved. Spontaneous PTD is a syndrome of numerous pathological processes (3), including certain maternal or fetal conditions, such as preeclampsia, intrauterine infection, cervical insufficiency, uterine anomalies, preterm premature rupture of membranes (PPROM), polyhydramnios, and fetal malformations (8). Previously observed risk factors also include, prior PTD, multifetal gestation, young or advanced maternal age, assisted reproductive technology (ART), black race, smoking, extremes of body-mass index (BMI), and low socioeconomic status (2).

Multiple lines of evidence support the role of maternal vaginal microbiota as a potential risk factor for PTD. Most notably, as much as 40–50% of premature deliveries are assumed to be due to maternal infection and inflammation (9–11). Prior research has shown that the most frequent cause of bacterial infection represents ascending pathway from the vagina through the cervix and into the uterus (3), where bacteria provoke deciduitis and chorioamnionitis or enter into the amniotic cavity and the fetus. Immediately after passing amniotic membranes, bacteria and their products are detected by pattern recognition receptors, such as toll-like receptors (TLRs), which causes the production of pro-inflammatory cytokines, chemokines, prostaglandins, and proteases leading to myometrial contractions. Additionally, inflammatory cascade leads to remodeling and disruption of fetal membranes (3, 11, 12). Hematogenous dissemination has also been proposed as an alternative route of intrauterine infection, as bacteria involved in periodontal disease have been found in the amniotic fluid (13). Furthermore, *in utero* microbial colonization of fetus has been suggested to have a role in the early establishment of the infant's microbiome (14, 15).

During pregnancy, a rise in the level of progesterone and estrogens, as well as immunological changes, significantly remodel composition of the human vaginal microbiome toward stable, low richness and diversity community structures dominated by *Lactobacillus* spp., which provide greater resistance and a protective role against genital tract infections (16, 17). Although dysbiosis (disruption of the vaginal microbiota) is asymptomatic in about 40% of affected women, it may predispose to a higher risk of urogenital conditions including aerobic vaginitis (AV) and bacterial vaginosis (BV) (18). The latest was reported to increase the risk of PTD ~5-fold (19).

The link between bacterial diversity and PTD has previously been demonstrated (20). Additionally, recent advances in the culture independent-techniques, using next-generation sequencing technologies proposed the causative role of overall vaginal microbiota composition in the development of PTD, although findings across studies have been inconsistent and largely ethnically dependent (7, 21–29). Therefore, the role of the vaginal microbiome in the etiology of PTD remains to be delineated. However, studies (7, 17, 25, 30) have unequivocally agreed that racial differences in the composition of vaginal microbiome play an important role in the detection of associations. Additionally, different conclusions about the role of vaginal microbiota in the development of PTD may have been reached due to the following reasons: (1) various definitions of PTD, (2) inclusion criteria varied widely (from low-risk PTDs to medically indicated and high-risk PTDs), (3) small power, (4) diverse designs, including different sequencing technologies and different 16S regions sequenced.

Although most of the previous 16S sequencing studies have identified a degree of association between the vaginal microbiome and PTD, there are inconsistencies in results that cannot currently be attributed to either different methodologies, heterogeneous definitions of PTD, or actual variations between study populations. Therefore, we used high-throughput 16S rRNA sequencing of the V3-V4 region in an independent Caucasian population to characterize whether the composition of maternal vaginal microbiota prior delivery differs between women who subsequently delivered preterm and those who delivered term.

## Materials and Methods

### Ethics Statement

All the women provided written informed consent prior to recruitment. This study was approved by the Slovenia National Medical Ethics Committee (approval number 90/02/15) and was conducted according to the principles expressed in the Declaration of Helsinki.

### Sample Collection and Clinical Assessment

We included 48 pregnant, Caucasian women, with spontaneous onset of premature labor with or without PPROM (≤36^6/7^ weeks) (cases) and 107 pregnant women with a spontaneous onset of labor with or without premature rupture of membranes (PROM) or planned cesarean section at term (≥38^0/7^) (controls). The women gave birth at the Department of Perinatology of the University Medical Center Ljubljana in the period from October 2015 to January 2019. We assessed the gestational age at delivery by last menstrual period and confirmed it through ultrasound examination in the first trimester by the measurement of the fetal crown-rump length (31). Vaginal swabs of the lateral wall of the vagina were collected by experienced obstetricians of the women who exhibited signs of labor based on the presentation of uterine contractions and cervical dilation at the admission to the delivery department.

Women who delivered preterm were subdivided into two groups according to the gestational age of labor; early preterm delivery (EPTD) (≤33^6/7^ weeks) and late preterm delivery (LPTD) (34^0/7^to 36^6/7^ weeks).

The inclusion criteria for both, cases and controls, were as follows: (1) healthy pregnant woman with healthy fetus, (2) singleton pregnancy, (3) spontaneous onset of labor with or without PPROM for cases and a spontaneous onset of labor with or without PROM (38^0/7^ to 40^6/7^ weeks) or a planned cesarean section between 39^0/7^ and 40^6/7^ weeks of gestation for controls (in the absence of exclusion criteria), (4) ≥18 years of age, and (5) ability to provide informed consent. Exclusion criteria for cases and controls were: (1) use of antibiotic within the week prior to inclusion, (2) use of vaginal antimicrobials, antibiotics or lactic acid <1 week prior to inclusion, (3) individuals receiving immunosuppressive therapy, (4) placenta previa, (5) uterine anomaly, (6) chronic disease, including pre-pregnancy hypertension, diabetes mellitus, and morbid obesity (class III WHO), (7) human immunodeficiency virus (HIV) or hepatitis C positive status, (8) bleeding due to abruption or placenta previa, (9) vaginal lavage during or before pregnancy, (10) hypertensive diseases in pregnancy (preeclampsia), (11) fetus anomalies, (12) intrauterine death, and (13) intrauterine growth restriction (IUGR).

National Perinatal Information System of Slovenia (NPIS) was reviewed to collect additional data on the pregnancy, delivery and postpartum. Women participating to this study were also asked to complete a brief in-person questionnaire to collect additional demographic and medical information (age, working status, cigarette smoking, pre-pregnancy BMI, antibiotics use during pregnancy, gestational diabetes, previous PTD, and vaginitis.

### Bacterial DNA Isolation, 16S rRNA Gene Amplification and Sequencing

The vaginal swabs for genomic bacterial DNA isolation were transferred to the laboratory in the Amies transport medium and stored at −80°C until isolation. The enzymatic and mechanical cell lysis was performed as described by Ravel et al. (30). The lysate was processed using the QIAamp DNA Mini Kit (QIAGEN, Hilden, Germany), according to manufacturer's guidelines for DNA purification from buccal swabs (recommendations for cotton or Dacron swabs) with minor modifications, including incubation at 70°C for 10 min instead of 56°C and 5-min incubation step at room temperature after the addition of 100 μl of heated (56°C) elution buffer. The DNA concentrations in the samples were quantified using the Qubit dsDNA high-sensitivity Assay Kit (Thermo Fischer Scientific). After isolation, the selected region (V3-V4) of the species-specific 16S rRNA gene was amplified by a polymerase chain reaction (PCR), using a universal set of Illumina adapter containing primers: 341F (5′-TCGTCGGCAGCGTCAGATGTGTATAAGAGACAG**CCTACGGGNGGCWGCAG**-3′) and 805R (5′-GTCTCGTGGGCTCGGAGATGTGTATAAGAGACAG**GACTACHVGGGTATCTAATCC**-3′). V3-V4 region was chosen to also assess differences in *Gardernella* abundance between groups (32). For the preparation of libraries, we used the standardized 16S Metagenomic Sequencing Library Preparation Protocol (Illumina®), using KAPA HiFi HotStart ReadyMix 2X for amplification. The amplicons of each sample were labeled with Nextera XT Indexes. The PCR products were quantified and quality checked by using Bioanalyzer DNA 1000 chip (Agilent Technologies, Santa Clara, CA, USA). Libraries of pooled samples were sequenced on the Illumina MiSeq sequencer according to the manufacturer's specifications in the 2 × 300 bp pair-end runs (MiSeq Reagent kit v3).

### Bioinformatics Analysis

The sequence data were imported into the R statistical environment (version 3.5.1) from demultiplexed FASTQ files, containing sequences with an average Phred score >30. Microbiome analysis was performed using Bioconductor software^1^ following Callahan et al. (33) workflow for Big Data. Trimming and filtering were carried out with The Divisive Amplicon Denoising Algorithm 2 (DADA2) (34) on paired reads jointly, with forward reads truncated at position 260 nt, and the reverse reads at position 240 nt based on the quality scores. First 17 nucleotides of forward reads and 21 nucleotides of reverse reads were trimmed to remove primers with ambiguous sequences. Filtering parameters were as follows; zero ambiguous base calls; enforcement of a maximum of 2/3 expected errors per forward/reverse reads, respectively. The relationship between quality scores and error rates was assessed separately for each run in order to minimize batch effects resulting from run-to-run variability. DADA2 was used to produce a sequence table by independently inferring amplicon sequence variants (ASVs) from the forward and reverse sequences of each sample using the run-specific error rates and to further merge read pairs. ASV represents a higher-resolution analog of the common operational taxonomic unit (OTU) table (34), such that it clusters the sequence reads into a finer taxonomic resolution without the need for a particular similarity cut-off (34, 35). Sequences identified as chimeric were removed. Taxonomy was assigned to ribosomal sequence variants using the RDP (Ribosomal Database Project) naive Bayesian classifier algorithm in the DADA2 R package and Silva v128 database (36). Only annotations that had more than 0.6 in their bootstrap confidence estimation values were accepted for further analyses. Clinical, taxonomic, AVS and phylogenetic data were combined into a single object using the phyloseq package (37) (version 1.24.2) for R. Due to the limitations of species-level classification with 16S rRNA sequencing, an additional aligning of representative sequences was performed by the Basic Local Alignment Search Tool (BLAST) (38), where necessary.

### Statistical Analysis

All statistical analyses were conducted using R programing language (version 3.5.1). Alpha-diversity metrics were calculated at ASV-level, after rarefying at the minimal sequencing depth of 39,998 reads by performing 1,000 iterations of random subsampling. Rarefaction analysis was performed to evaluate whether further sequencing would likely detect additional AVSs, using the vegan^2^ package for R. The microbial richness (number of unique ASVs) was evaluated by the Chao1 index, and alpha diversity by Shannon diversity index, which takes into account richness as well as the abundance of ASVs for each sample, using the phyloseq package (version 1.24.2) (37). Continuous variables were tested for the normality with the Shapiro-Wilk normality test and visually inspected by histograms. Differences in richness and diversity between the different sample groups were calculated by the nonparametric Wilcoxon rank-sum test and the Kruskal-Wallis test when comparing three groups (39).

To estimate differences in beta-diversity, ASVs occurring in fewer than 3 samples and with a count of <10 across all samples were excluded from raw data. The ASV table normalized to relative abundance values and multidimensional scaling (MDS) was performed on phylogenetic distance matrices; weighted UniFrac (40). Variation in the overall microbial community structure between groups was assessed with permutational multivariate analyses of variance (PERMANOVA), using *adonis* function implemented in the R package vegan with 999 permutations for significance testing. Differences between the outcome groups were visually presented by generating Principal Coordinates Analysis (PCoA) plots and confidence ellipses in the R package ggplot.

The differential relative abundance of taxa between groups was determined at ASVs level using the Bioconductor package DESeq2 (version 1.20.0) in R (41). After variance-stabilizing transformations, we normalized samples using the nonparametric Wald negative binomial regression. Results were expressed as log2 fold change and taxa were considered significantly differentially abundant between groups if their adjusted *P*-value was <0.05. ASVs with prevalence >3% and >50 reads study-wide were included in the DESeq2 analysis.

Vaginal community state types (CST) were determined at ASV level, following the script by DiGiulio et al. (24). The Bray-Curtis dissimilarity metric, based on relative abundance was used to calculate the distance between all samples. Medoid clustering approach (*pam* in R) was applied to PCoA distances. The appropriate number of clusters was determined using the gap statistic (*k* = 5) (42).

For hypothesis testing of specific-taxa associations, we performed one-sided Wilcox rank-sum tests on the mean relative abundance of a taxon across samples from the same group. *P*-values were adjusted for multiple comparisons using the Benjamini-Hochberg false-discovery rate (FDR) correction method (43).

## Results

### Participants Characteristics

Vaginal microbiome sequencing was performed in 158 Caucasian pregnant women who contributed vaginal swabs at the admission to the perinatal delivery department. Three cases were excluded after the medical records were reviewed because the PTD was indicated due to IUGR. The majority of the participants had a vaginal delivery (94.8%), except eight controls, who delivered with a planned C-section (5.2%). Table 1 reports demographic and clinical features of participants. Of the included 155 women, 48 (31.0%) subsequently experienced PTD and 34 (70.8%) of these PTDs were classified as EPTD (<34^0/7^ weeks.). Women who delivered at term did not differ significantly from those who delivered preterm in terms of age, working status, parity, pre-pregnancy BMI, the presence of thyroid disease, gestational diabetes mellitus (GDM), presence of asymptomatic bacteriuria, presence of vaginitis and vaginal treatment during pregnancy, urogenital infection, and smoking status (Table 1). All of the included participants were Caucasians of Slovenian ethnicity.

**Table 1:** Demographic and clinical characteristics of participants.

**Demographic and clinical characteristics** **Total (*n* = 155)**	**Preterm ≤36^**6/7**^weeks** ***n* = 48 (%)**	**Term ≥38^**0/7**^ weeks** ***n* = 107 (%)**	***P*-value, **χ^2^**-test,** **^a^Wilcoxon rank-sum test**
**Maternal age at delivery**, **Years; Mean ± SD**	31.40 ± 4.73	30.93 ± 4.13	^a^*P* = 0.235
**Pre–pregnancy BMI (kg/m**^**2**^**), Mean** **±** **SD**	24.17 ± 4.98	23.34 ± 4.09	^a^*P* = 0.485
Underweight (<18.5)	4 (8.3)	8 (7.5)	*P* = 0.284, χ^2^ = 3.80
Normal Weight (18.5–24.9)	30 (62.5)	63 (58.9)	
Overweight (25.0–29.9)	7 (14.6)	28 (26.2)	
Class I and II Obesity (30.0–39.9)	7 (14.6)	8 (7.5)	
**Gestational age at delivery (weeks; mean** ± **SD)**	30.94 ± 3.79	40.03 ± 1.40	^a^***P*** **<** **0.00001**
**Birthweight (g; mean** ± **SD)**	1676.96 ± 733.54	3511.31 ± 376.44	^a^***P*** **< 0.00001**
**Gestational Diabetes mellitus**			
Yes/No	9 (18.75)/39 (81.25)	11 (10.3)/96 (89.7)	*P* = 0.1459, χ^2^ = 2.12
**Working status**			
Employed/Unemployed	39 (81.25)/9 (18.75)	86 (80.4)/21 (19.6)	*P* = 0.898, χ^2^ = 0.0163
**Smoking**			
Yes	7 (14.6)	10 (9.3)	*P* = 0.489, χ^2^ = 1.43
No	40 (83.3)	92(86.0)	
Quit before pregnancy	1 (2.1)	5 (4.7)	
**Thyroid disease**			
Yes/No	4 (8.3)/44 (91.7)	8 (7.5)/99 (92.5)	*P* = 0.854, χ^2^ = 0.034
**Progesterone therapy**			
Yes/No	16 (33.3)/32 (66.7)	13 (12.1)/94 (87.9)	***P*** **=** **0.00177**, **χ^2^** **=** **9.78**
**TOCOLYTIC (ATOSIBAN)**			
Yes/No	5 (10.4)/43 (89.6)	0	
**Vaginitis and treatment**			
Yes/No	16 (33.3)/32 (66.7)	45 (42.1)/62 (57.9)	*P* = 0.304, χ^2^ = 1.06
**Urogenital infection**			
Yes/No	5 (10.4)/43 (89.6)	7 (6.5)/100(93.5)	*P* = 0.404, χ^2^ = 0.697
**Asymptomatic bacteriuria**			
Yes/No	1 (2.1)/47 (97.9)	2 (1.9)/105 (98.1)	*P* = 0.929, χ^2^ = 0.008
**Antibiotic use during gestation**			
Yes/No	9 (18.75)/39 (81.25)	12 (11.2)/95(88.8)	*P* = 0.205, χ^2^ = 1.61
**Parity**			
0	24 (50.0)	42 (39.3)	*P* = 0.305, χ^2^ = 2.37
1	20 (41.7)	48 (44.9)	
2-4	4 (8.3)	17 (15.9)	
**Prior preterm delivery**			
Yes/No	5 (10.4)/43 (89.6)	3 (2.8)/104 (97.2)	***P*** **=** **0.0476**, **χ^2^** **=** **3.92**

*Distributions of categorical variables are compared using Pearson chi-square test at a 5% level of significance and distribution of continuous variables with non-normal distribution with Wilcoxon rank-sum test. Characteristic with significant differences (at 5% level) between preterm and term cohorts are in bold*.

As reported previously, the women with prior PTD history were more likely to experience subsequent PTD (*P* = 0.0476). Of the PTD group, 18 women (37.5%) experienced PPROM. As expected, the PTD group had a greater number of women, receiving progesterone therapy (*P* = 0.00177). None of the women reported vaginal intercourse during the 3 days before sampling.

### 16S Sequencing Results

A total of 155 swab samples were analyzed, comprising of 48 PTD samples and 107 controls. After initial quality control steps and chimera removal, Illumina MiSeq sequencing of 16S rRNA gene resulted in a total of 12,233,991 processed paired-end V3-V4 reads, with an average read count of 78,928.97 reads per sample (min = 39,998; max = 223,503) (Supplementary Figure S1A).

Next, rarefaction curves were generated by subsampling evenly to the minimal depth of 39,998 reads. Rarefaction analysis showed that curves for each sample reached the plateau and thus no additional ASVs were expected to be found by increasing sequencing depth (Supplementary Figure S1B).

After removal of singletons, doubletons, rare ASVs (defined as prevalence ≥0.018 (in <3 samples), uncharacterized phylum, and taxa with total reads of <10 (8 × 10^−7^) study-wide, 390 ASVs were identified across 155 samples. 11,984,382 reads remained, presenting 94.2% of the total dataset.

### Alpha and Beta Diversity

**Figure 1:**
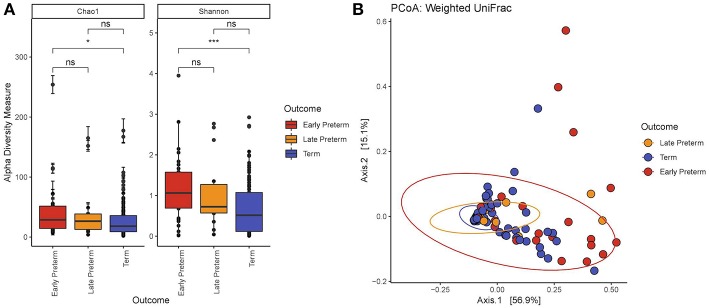
**(A)** Alpha diversity by the gestational delivery group. Chao1: Kruskal-Wallis χ2 = 6.76, *P* = 0.0034; Shannon: Kruskal-Wallis χ2 = 12.4, *P* = 0.0021. The microbiome of EPTD group has increased richness (Chao1) and diversity (Shannon Index) compared to term participants. **(B)** Principal coordinate analysis (PCoA) plot created at the ASV level generated by the Weighted Unifrac distance. The values in parentheses show the percentages of total community variation explained. The red dots represent EPTD women, the orange dots represent LPTD women and the blue dots represent women who delivered at term. *P*-values <0.05 and <0.001 are represented as ^*^ and ^***^, respectively.

We demonstrated that the microbiome of participants across EPTD, LPTD, and term gestational delivery groups differed significantly in richness (Figure 1A) (Chao1, Kruskal-Wallis, *P* = 0.034). Individual comparison using Wilcoxon rank sum test showed that Chao1 index decreased in the term group compared to the all preterm cases (Chao1*, P* = 0.011) (Supplementary Figure S2) or EPTD group (Chao1*, P* = 0.012), indicating the reduced richness in women who delivered term babies.

Comparison of bacterial diversity between EPTD, LPTD, and term, revealed significant differences by Shannon index in the Kruskal-Wallis test (*P* = 0.0021) (Figure 1A). By Wilcoxon rank sum test we demonstrated a significant decrease in diversity in the term group compared to all preterm cases (Shannon, *P* = 0.00059) (Supplementary Figure S2A) or EPTD group (Shannon*, P* = 0.00079).

Next, we independently tested PPROM for alpha diversity, comparing cases who experienced PPROM (*n* = 18) to cases who delivered spontaneously without PPROM (*n* = 30). Among mothers with PTD, those who experienced PPROM showed a trend toward greater richness when compared to spontaneous PTD cases without PPROM, although the results were not statistically significant (Wilcoxon test, Chao1, *P* = 0.097) (Supplementary Figure S3A).

With respect to beta diversity, weighted UniFrac analysis demonstrated that overall microbial community composition of vaginal swabs was significantly different by delivery gestational age category (Figure 1B). This was statistically confirmed with the non-parametric adonis test (*P*_WeightedUniFrac_ <0.001) (Table 2). Figure 1B shows the two-dimensional principal coordinate analysis (PCoA) plot, which indicates a clustering of EPTD, LPTD, and term groups, away from each other.

**Table 2:** Results from adonis (PERMANOVA) analysis (UniFrac distance and similarity measurements).

**Comparison**	**Weighted unifrac distance**	
	***R^**2**^* value**	***P*-value**
PTD vs. Term (155 samples) *n* = 48, *n* = 107	0.0818	<0.001
EPTD vs. Term (141 samples) *n* = 34, *n* = 107	0.106	<0.001
Global effect, EPTD, LPTD, Term*, df* = 2 (155 samples) *n* = 34, *n* = 14, *n* = 107	0.0970	<0.001

*BMI, body mass index; EPTD, early preterm delivery; LPTD, late preterm delivery; PPROM, premature rupture of membranes; PTD, preterm delivery; SD, standard deviation*.

We did not detect significant differences in beta diversity between preterm cases who experienced PPROM and preterm cases who did deliver spontaneously without PPROM (Supplementary Figure S3B).

### Composition of the Vaginal Microbiome

**Figure 2:**
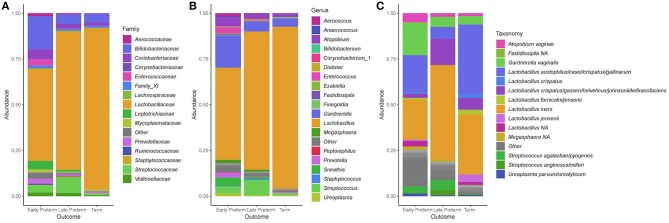
Stacked bar plots of average relative abundance at the **(A)** family, **(B)** genus, and **(C)** species taxonomic levels compared according to the gestational age of delivery. *Lactobacillus acidophilus/casei/crispatus/gallinarum*: 100% *L. crispatus*/100% *L. gallinarum* according to BLAST; *Lactobacillus crispatus/gasseri/helveticus/johnsonii/kefiranofaciens*: 100% *L. gasseri* according to BLAST.

Across 155 participants, the dominant phyla in the vaginal microbiota were Firmicutes and Actinobacteria, which respectively made up 84.4 and 10.8% of total abundance, with lower contributions from Fusobacteria (1.29%), Proteobacteria (0.902%) and Tenericutes (0.874%). At order level, the top five dominant taxa were Lactobacillales (81.9%), Bifidobacteriales (8.07%), Coriobacteriales (2.48%), Fusobacteriales (1.29%), and Enterobacteriales (0.671%). The predominant bacterial families were *Lactobacillaceae* (77.9%), *Bifidobacteriaceae* (8.07%), *Coriobacteriaceae* (2.48%), *Streptococcaceae* (2.57%), and *Leptotrichiaceae* (1.23%) (Figure 2A). At the genus level, the dominant members were *Lactobacillus* (77.9%), *Gardnerella* (7.73%), *Atopobium* (2.46%), *Streptococcus* (2.57%), and *Sneathia* (1.20%) (Figure 2B). At the species level, *L. iners* and *L. crispatus* were most abundant (Figure 2C).

In women, who delivered at term, the *Lactobacillus* genus constituted 88.3% and *Gardnerella* 4.63% of total bacteria. In that group 91/107 (85%) samples had *Lactobacillus* abundance above 70%, and 55/107 (51%) samples harbored *Lactobacillus* genera above 98%. In the EPTD group, *Lactobacillus* comprised 50.3% and *Gardnerella* 17.6%, and in LPTD 75.5 and 5.22%, respectively. *Atopobium* was present at higher percentages in the EPTD (5.05%) and LPTD (2.13%) groups when compared to the term group (1.59%). Relative abundances of genera within each sample according to a week of delivery are presented in Supplementary Figure S4.

### Differential Abundance at ASVs Level Between Delivery Groups

Hypothesis-free approach at ASV level, using differential abundance analysis (DESeq2) revealed more than 24-fold higher abundance of ASVs belonging to species *Lactobacillus gasseri* and more than 6-fold higher abundance of ASVs belonging to *Lactobacillus jensenii* in women who delivered term compared to women who delivered preterm. 8-fold and 7-fold higher abundance of ASVs belonging to *Sneathia sanguinegens* and *Streptococcus agalactiae/pyogenes* were detected in the PTD group when compared to term (Supplementary Figure S5A). When EPTD and term groups were compared, results additionally indicated a higher abundance (3-fold) of *Lactobacillus crispatus/gasseri/helveticus/johnsonii/kefiranofaciens* (100% *L. gasseri* according to BLAST) in the term group (Supplementary Figure S5B). The AVSs representing the following taxa were detected differentially abundant in preterm vs. term comparison (*P*_*unadj*_ <0.05), bud did not reach the significance after FDR adjustment for multiple testing: *Lactobacillus crispatus/gasseri/helveticus/johnsonii/kefiranofaciens* (100% *L. gasseri* according to BLAST), *Shewanella algae/indica, Prevotella disiens, Gardnerella vaginalis, Streptococcus anginosus/milleri, Prevotella bivia/denticola*, and *Enterococcus faecalis*. *Prevotella timonensis, Anaerococcus hydrogenalis, Lactobacillus acidophilus/casei/crispatus/gallinarum* (100% *L. crispatus*/*L. gallinarum*, BLAST), *Atopobium vaginae, Staphylococcus haemolyticus/petrasii*, and *Bifidobacterium longum* were detected as marginally significant before FDR adjustment (Supplementary Tables S1, S2).

### Relative Abundance Comparisons of Individual Taxa

At the genus level, we analyzed and confirmed the reduced mean relative abundance of *Lactobacillus* in the PTD group when compared to term (*P*_*adj*_ = 0.000177), using one sided-Wilcoxon rank-sum test on taxonomically grouped data.

Furthermore, we tested for some of the additional previously reported associations between PTD and genera observed to have increased abundance during BV or AV (24, 25) (Table 3). At the genus level, significant associations for following taxa, previously associated with BV were confirmed in our dataset: *Gardnerella, Atopobium, Sneathia, Gemella, Megasphaera*, and *Dorea*. We did not detect significant associations with PTD in mean relative abundances of the following genera: *Ureaplasma, Mycoplasma, Mobiluncus*, and *Peptoniphilus*. Among genera associated with AV, we detected an increased abundance of *Streptococcus* and *Escherichia/Shigella* (Table 3).

**Table 3:** Hypothesis testing: The *P*-values were calculated with the one-sided Wilcoxon rank-sum test and then adjusted for multiple hypotheses by the Benjamini–Hochberg method.

**Genus level**	**Hypothesis**	**Comparison** **(*n* = 48, *n* = 107)**	***P*-value**	***P*-adjusted** **(FDR)**	**Significance**
*Lactobacillus*	Decreased	Preterm-Term	0.0000142	0.000177	^***^
**BV**					
*Prevotella*	Increased	Preterm-Term	0.0874	0.124	ns
*Gardnerella*	Increased	Preterm-Term	0.0153	0.0324	^*^
*Atopobium*	Increased	Preterm-Term	0.0000850	0.000482	^***^
*Sneathia*	Increased	Preterm-Term	0.0000209	0.000177	^***^
*Gemella*	Increased	Preterm-Term	0.00550	0.0187	^*^
*Megasphaera*	Increased	Preterm-Term	0.00434	0.0184	^*^
*Ureaplasma*	Increased	Preterm-Term	0.327	0.427	ns
*Mycoplasma*	Increased	Preterm-Term	0.474	0.474	ns
*Mobiluncus*	Increased	Preterm-Term	0.453	0.474	ns
*Dialister*	Increased	Preterm-Term	0.0711	0.121	ns
*Peptoniphilus*	Increased	Preterm-Term	0.394	0.447	ns
*Dorea*	Increased	Preterm-Term	0.00925	0.0262	^*^
**AV**					
*Streptococcus*	Increased	Preterm-Term	0.0132	0.0321	^*^
*Staphylococcus*	Increased	Preterm-Term	0.377	0.447	ns
*Enterococcus*	Increased	Preterm-Term	0.0877	0.124	ns
*Escherichia/Shigella*	Increased	Preterm-Term	0.0249	0.0471	^*^
**Species level**	**Hypothesis**	**Comparison** **(*****n*** **=** **48**, ***n*** **=** **107)**	***P*****-value**	***P*****-adjusted** **(FDR)**	**Significance**
*L. crispatus*^a^	Decreased	Preterm-Term	0.0201	0.0402	^*^
*L. iners*	Increased	Preterm-Term	0.312	0.312	ns

Adjusted p <0.05 was considered significant. P-values <0.05 and <0.001 are represented as

* and

*** *, respectively. BV, bacterial vaginosis; AV, aerobic vaginitis; FDR, false discovery rate*.

a *Lactobacillus acidophilus/casei/crispatus/gallinarum: 100% L. crispatus/100% L. gallinarum according to BLAST*.

At the species level, we individually tested for *L. crispatus* and *L. iners* associations with PTD. The mean relative abundance of ASV belonging to *Lactobacillus acidophilus/casei/crispatus/gallinarum* (classified as 100% *L. crispatus*/*L. gallinarum* according to BLAST) was enriched in the term group compared to PTD (one-sided Wilcox-test, *P*_adj_ = 0.0201), with 38.1% in the term and 26.9% in the PTD group. Association of *L. iners* with gestational age delivery group was not significant.

### Vaginal Microbiome Community State Types (CSTs)

**Figure 3:**
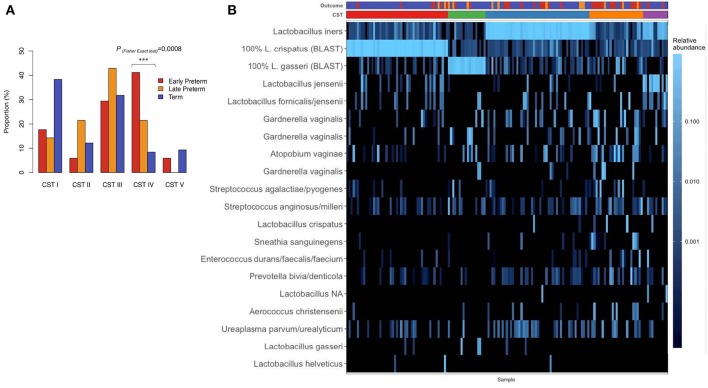
**(A)** The proportion of delivery group; early preterm (*n* = 34), late preterm (*n* = 14), term (*n* = 107), in specific CST. Significant difference was detected in overall frequency of five CSTs between term, LPTD, and EPTD (*P*_*Fisher exact test*_= 0.0025) and between term-EPTD groups (*P*_*Fisher exact test*_= 0.0005). **(B)** The heatmap of the relative abundance of 20 most abundant ASVs in the vaginal microbiome before the delivery of pregnant women by early preterm, late preterm, and term delivery. Frequencies of CSTI, CST II; III; IV; V in the entire sample were: 31.6, 11.6, 32.3, 16.8, 7.74%. *P*-value <0.001 is represented as ^***^.

Hierarchical clustering analysis demonstrated that vaginal microbiota clustered into 5 distinct community profiles, consisting of *L. crispatus* (CST I), *L. gasseri* (CST II), *L. iners* (CST III), and *L. jensenii/L. iners*-dominated clusters (CST V) and a cluster depleted of *Lactobacilli* species and enriched for anaerobic bacteria (CST IV) (Figure 3). Frequencies of specific CSTs in the entire sample were as follows: CST I (31.6%), CST II (11.6%), CST III (32.3%), CST IV (16.8%), and CST V (7.74%). In CST IV cluster were 41.2% of EPTD participants, 21.4% of LPTD participants, and only 8.41% of the term participants (Figure 3A). CST I (dominated by *L. crispaus*) was present in 38.3% of term delivery group, 17.6% in EPTD and 14.3% in the LPTD group (Figure 3A). We observed a significant difference in the overall frequency of five CSTs between three delivery groups (term, LPTD, and EPTD), *P*_*Fisherexacttest*_ = 0.0025, which was even more profound when only term and the EPTD groups were considered (*P*_*Fisherexacttest*_ = 0.0005).

When we tested previously reported association of *Lactobacillus*-depleted CST IV with PTD, the CST IV was significantly overrepresented in the EPTD group compared to term (*P*_Fisher exact test_ = 0.0008).

## Discussion

Our findings showed that pregnant women who delivered preterm relative to term have higher richness and diversity of the vaginal microbiome, along with reduced *Lactobacillus* species, especially reduced *L. crispatus* and the increased abundance of the genus *Gardnerella*. Additionally, we found that overall frequencies of CSTs were significantly different between EPTD, LPTD, and term delivery groups, with CST IV (enriched for BV-associated taxa and higher diversity) observed in the EPTD group more often than in the term group. These observations are in accordance with the majority of the previously published 16S sequencing studies investigating the similar research question in Caucasian populations.

The higher alpha diversity and lower abundances of *Lactobacillus* spp. (CST IV) in the early stages of pregnancy were previously correlated with gestational age at delivery (24, 25). CSTs were previously shown to differ between term and preterm deliveries also in the study of Stafford et al. (27), where PTD has been associated with CST V (*L. jesenii*) dominance, while CST I (*L. crispatus*) and CST II (*L. gasseri*) were beneficial for term delivery. The association between PTD and vaginal dysbiosis (CST IV) was not confirmed in the study of Kindinger et al. (22). This discrepancy may be explained by the inclusion of only women at high-risk for PTD, possibly making the microbial-cause for PTD less prominent in that cohort. The study of Freitas et al. (44), conducted in Canadian cohort also failed to detect specific vaginal microbiome CSTs early in pregnancy that could predict PTD, however, the findings of increased richness and diversity in PTD are in agreement with our study, despite different methodologies used (pyrosequencing of cpn60).

While the protective role of *L. crispatus* was repeatedly demonstrated in most previous 16S sequencing studies (21, 22, 25, 27, 29), association of *L. iners* with PTD yielded conflicting results. The largest 16S sequencing study of white European population investigating the role of vaginal microbiota in PTD (21), concluded that *L. iners/Ralstonia solanacearum* oligotype was associated with decreased risk of early (<34 weeks), but not late (34–36 weeks) PTD, as well as *L. gasseri/L. johnsonii, L. crispatus/L. acidophilus*, and *Bifidobacterium longum/Bifidobacterium breve* oligotypes. In contrast, a recent 16S sequencing study of V1-V3 region by Kindinger et al. (22) showed that dominance of *L. iners* at 16 weeks of gestation is a potential risk factor for early PTD (<34 weeks). We did not manage to confirm the association with *L. iners* with either term or preterm delivery, however, our results indicated that its mean relative abundance was the highest in the LPTD group (51.8%), while it was more prevalent in term group (32.6%) compared to EPTD (21. 9%).

Consistent with the previous reports of an association between BV and AV with PTD (24, 25, 29), we detected increased abundance of BV-associated taxa, including *Gardnerella, Atopobium, Sneathia (Sneathia sanguinegens), Gemella, Dorea*, and AV-associated bacteria, *Streptococcus* (*Streptococcus agalactiae/pyogenes*), and *Escherichia/Shigella* genera in the PTD group, when compared to term. Contrary to the study of Callahan et al. (25), we did not confirm the association of genera *Mobiluncus, Peptoniphilus*, and *Mycoplasma* with PTD; however, we did detect the positive association of *Megasphaera* with PTD, which was not confirmed in a before-mentioned study. The association of *Ureaplasma* with PTD (24) was not replicated in our cohort, nor was it in the studies of Callahan et al. (25) and Freitas et al. (44). Similarly to our study, Elovitz et al. (7) reported the significant association of *Atopobium vaginae* with increased rates of PTD in non-Afro-American (AA) women, while *Sneathia, Atopobium*, and *Megasphaera* were associated with PTD when all women (AA and non-AA) were considered.

Our results differ from the results of the studies by Stout et al. (26) and Romero et al. (23), where no specific bacterial taxa or community was correlated with pregnancy outcome. Nevertheless, indicated PTDs (preeclampsia, fetal indications) were not excluded from the first study and only taxa that were present in at least 25% of samples were considered in the differential abundance analysis in the study of Romero et al. (23). We suppose that geographical and cultural differences (predominantly AA population), as well as a low number of PTD cases included (*n* = 23 and *n* = 18, respectively), might also contribute to negative results of those two studies.

Decreased *Lactobacilli* abundance manifests in the reduced lactic acid, hydrogen peroxide, and bacteriocins production, which enhances the proliferation of pathogenic bacteria known to cause BV (45, 46). Additionally, increased bacterial diversity has previously been associated with mucus and cytoskeleton alterations, cell death, increased expression of pro-inflammatory cytokines (29) and metalloproteinases and decreased IgG1/2 (47), which potentially leads to the inflammatory activation and stimulation of PTD. Furthermore, in the recent study, women with a non-*Lactobacillus* spp.-dominant microbiome were more likely to develop premature cervical dilation (48). It was also demonstrated (28, 49) that PPROM, which precedes 30% of all PTDs, is associated with high bacterial diversity and reduced *Lactobacillus* abundance prior to rupture. In the present study, we detected a tendency toward increased richness when preterm cases with PPROM were compared with preterm cases who delivered spontaneously without PPROM, but the result did not reach statistical significance.

It is already known that 16S sequencing experimental design impacts sensitivity to different taxa, and here we used the V3-V4 primer set, which is also sensitive to *Gardnerella*. Consequently, in our dataset *Gardnerella* represented 7.7% of the genus relative abundance, which is similar to study of the Callahan et al. (25) (5% of reads). The higher relative abundance of *L. iners* in the PTD group in the study of Kindinger et al. (22) may, therefore, be due to undetected *Gardernella* (V1-V3 primer set), which was shown to often coexist at high frequencies with *L. iners* (25), rather than a direct association of *L. inners* with PTD. Another explanation is that increased *L. iners* abundance during the 16th week of gestation might be associated with increased bacterial instability later in pregnancy, as *L. iners* is often associated with BV-related vaginal bacteria, due to its possible lack of protection against pathogens (50).

In contrast, *L. crispatus* has the largest genome of four *Lactobacilli* spp. (45), including genes encoding for bacteriocin, adhesin (anti-adhesive molecules against *Gardnerella* vaginalis), lactate, and succinate (51) and was reported to have protection against inflammation induced PTD (51). It has also been demonstrated that *D-lactic* acid levels were significantly higher when *L. crispatus* was the dominant vaginal bacterium compared to *L. iners* or *Gardernella* dominated vaginal microbiota. It was demonstrated that *D-lactic* acid enhances activation of anti-microbial innate and acquired immune system and also minimizes matrix metalloproteinase-8 (MMP-8) levels that would otherwise promote proteolytic activity and thus enhance bacterial migration past endocervix into the uterus, leading to infection-related PTD (52).

The strength of the present study is the inclusion of a large number of early PTDs (<34 weeks) compared to previous reports. Furthermore, we utilized strict inclusion criteria, meaning that PTDs indicated due to maternal or fetal complications, such as IUGR, preeclampsia, or fetal anomalies were excluded from the study. With an aim to exclude potential pre-existing risk factors for PTD and factors that could independently influence on microbiota composition, we also excluded individuals with chronic diseases, such as hypertension, diabetes mellitus, or women with the uterine anomalies. Another strength is the homogeneity of the study population, while it is known that microbiota composition is largely influenced by ethnicity (7, 17, 30). All participants were Caucasians and belonged to the same Slovenian ethnicity. Furthermore, compared to the most studies, we utilized bioinformatic approach with improved resolution and comprehensiveness that resolves exact ASVs, instead of imposing the arbitrary dissimilarity thresholds that define molecular OTUs (35).

Our study also has some limitations that need to be addressed. Firstly, sampling was conducted at a single time point; longitudinal sampling through pregnancy could add information about microbiome stability. Although previous studies showed that vaginal microbiome in the pregnancy remains relatively stable (24, 53), the increased *Lactobacillus* (23) and significant decrease in abnormal vaginal colonization rate were reported as the pregnancy progressed (54). Another limitation of our study is that 16S-sequencing technology does not assess the total abundance load of the taxa in the sample (gene copy measurement) that could be detected using a broad-range 16S rRNA quantitative PCR. Immune factors, that were suggested to lower the risk of PTD associated with vaginal microbiota, such as beta-defensin-2 (7) or proinflammatory cytokines (29), were not measured.

With a more comprehensive understanding of the vaginal microbiota in pregnancy in different populations, and its effect on PTD, detection of mothers at high-risk and development of pharmacological interventions for PTD prevention will become plausible.

In conclusion, our results suggest that maternal vaginal microbiome has an important role in the PTD etiology. In our independent analysis, we confirmed that vaginal microbiome of women who delivered preterm has increased richness and diversity prior delivery, decreased *Lactobacillus* abundance and the increased abundance of BV-associated taxa. Nevertheless, the role of vaginal microbiota in the etiology of PTD remains to be elucidated in further research considering different ethnicities and ideally with the integration of hosts and fetus genetic data.

## Data Availability

Generated Statement: The datasets generated for this study can be found in the Sequence Read Archive (NCBI) repository, BioProject PRJNA544732, http://www.ncbi.nlm.nih.gov/bioproject/544732.

## Ethics Statement

The studies involving human participants were reviewed and approved by Slovenia National Medical Ethics Committee, Ministry of Health, Štefanova 5, SI-1000 Ljubljana, Slovenia (approval number 90/02/15). The patients/participants provided their written informed consent to participate in this study.

## Author Contributions

KH, MV, TP, and AHo collected the samples. KH performed sequencing experiments and analyzed the data. BP, TP, and KH conceived and designed the study. KH wrote the manuscript. All authors contributed to the manuscript revision and approved the final manuscript.

### Conflict of Interest Statement

The authors declare that the research was conducted in the absence of any commercial or financial relationships that could be construed as a potential conflict of interest.

## Footnotes

^1^https://bioconductor.org

^2^https://CRAN.R-project.org/package=vegan

## Abbreviations

AA: African American
ART: assisted reproductive technology
ASV: amplicon sequence variant
AV: aerobic vaginitis
BLAST: Basic Local Alignment Search Tool
BMI: body-mass index
BV: bacterial vaginosis
CST: community state type
DADA2: Divisive Amplicon Denoising Algorithm 2
EPTD: early preterm delivery
FDR: false-discovery rate
GDM: gestational diabetes mellitus
GLM: generalized linear model
HIV: human immunodeficiency virus
IUGR: intrauterine growth restriction
LPTD: late preterm delivery
MDS, multidimensional scaling, MMP-8: matrix metalloproteinase-8
OTU: operational taxonomic unit
PBS: phosphate-buffered saline
PCoA: Principal Coordinates Analysis
PCR: polymerase chain reaction
PERIS: Perinatal Information System of Slovenia
PERMANOVA: permutational multivariate analyses of variance
PPROM: premature rupture of membranes
PROM: premature rupture of membranes
PTD: preterm delivery
RDP: Ribosomal Database Project
rRNA: ribosomal RNA
spp: species
TLRs: toll-like receptors
WHO: World Health Organization.
