# Supplementary material for: Vaginal Microbiome Signature Is Associated With Spontaneous Preterm Delivery
Source: Front Med (Lausanne). 2019 Sep 10;6:201. doi: 10.3389/fmed.2019.00201 (PMC6746969; doi:10.3389/fmed.2019.00201)
Supplement: Supplementary file 2 [file Data_Sheet_1.PDF]

Supplementary Material

1 Supplementary figures

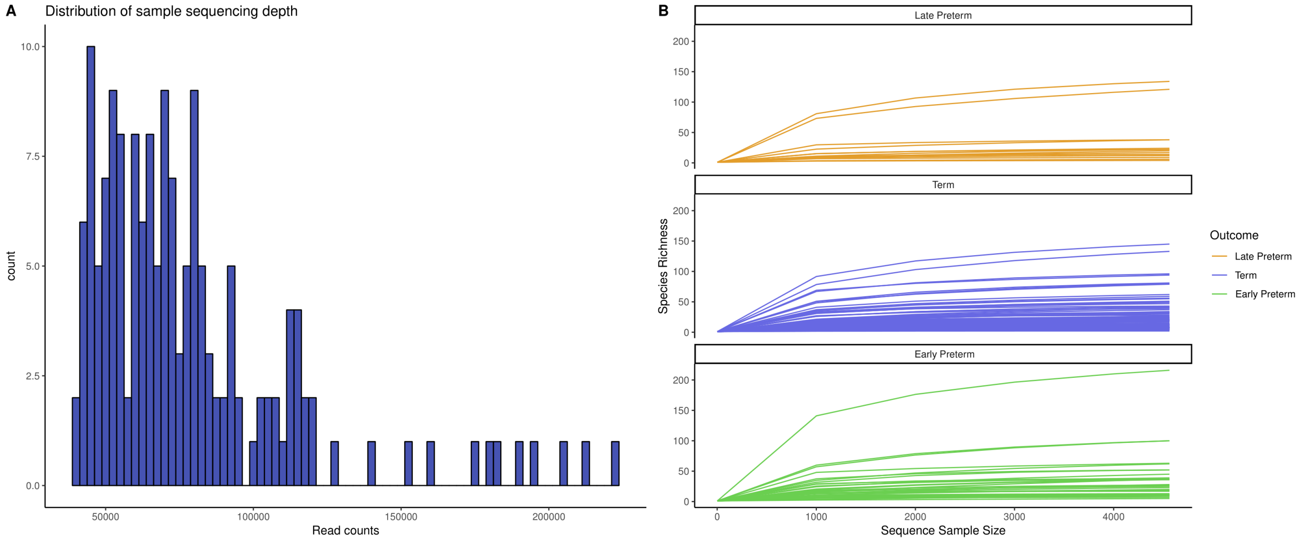

Supplementary Figure S1. (A) Distribution of sample sequencing depth. **(B)** Rarefaction analysis showed that curves for each sample reached the plateau and thus no additional ASVs were expected to be found by increasing sequencing depth. ASV, amplicon sequence variant.

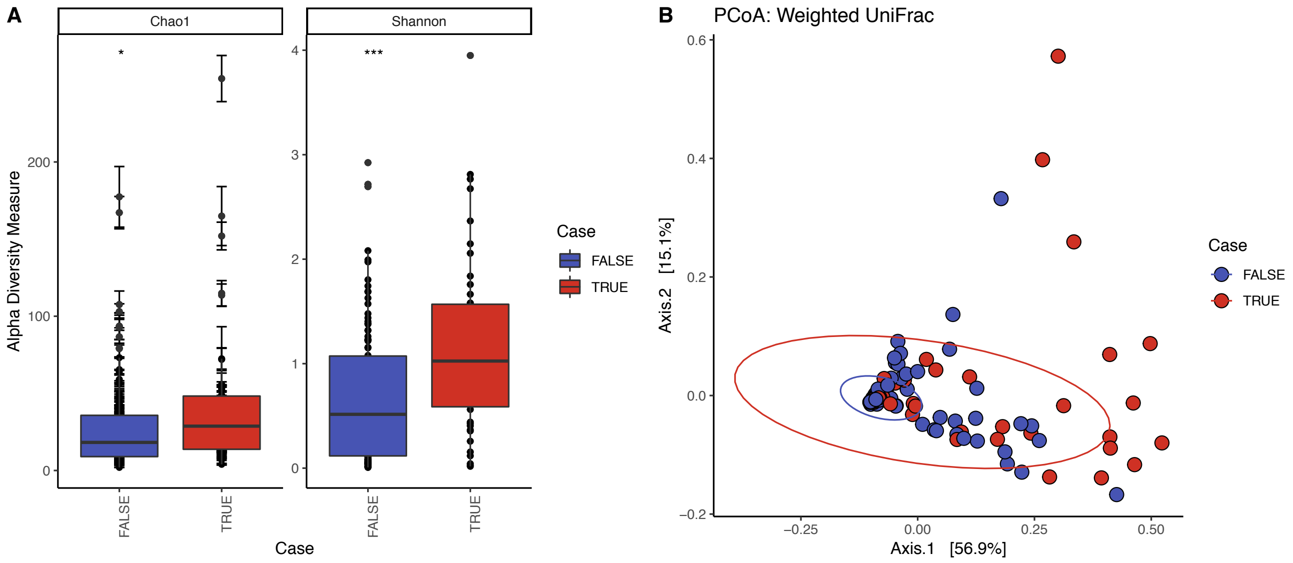

Supplementary Figure S2. **(A)** Alpha diversity by the gestational delivery group. The microbiome of the preterm group has increased richness (Chao1, P=0.011) and diversity (Shannon, P=0.00059) compared to term participants. **(B**) Principal coordinate analysis (PCoA) plot created at the ASV level generated by the Weighted Unifrac distance ($P_{\text{Weighted UniFrac}}$<0.001), where the values in parentheses show the percentages of total community variation explained. The red dots represent preterm women and the blue dots represent women who delivered at term. ASV, amplicon sequence variant.

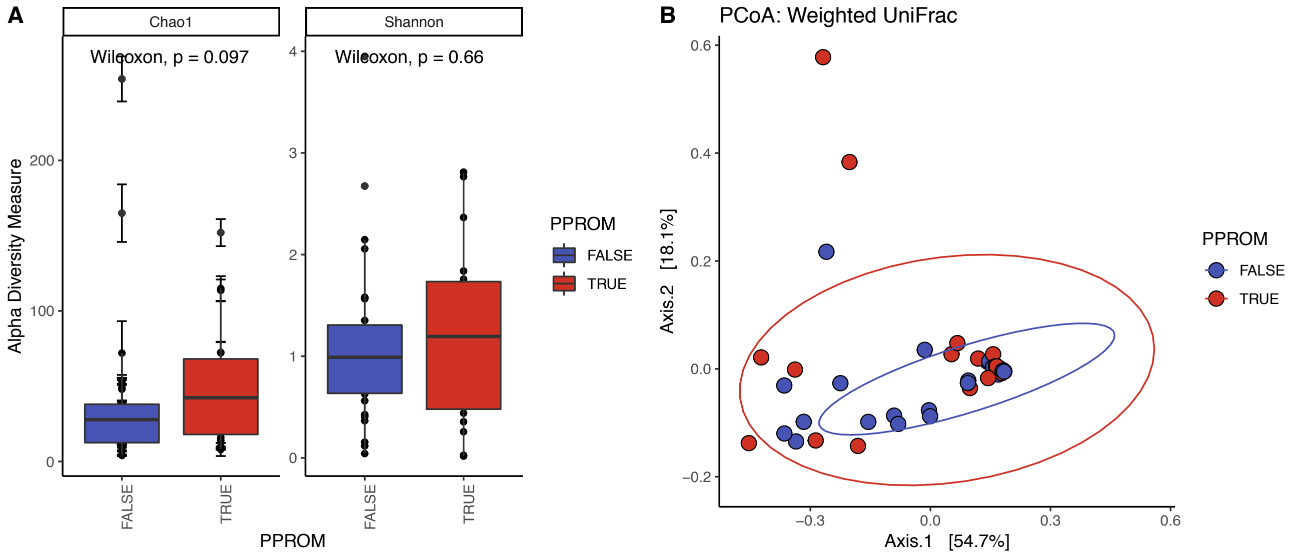

Supplementary Figure S3.

(A) Alpha diversity by preterm cases with preterm premature rupture of membranes (PPROM) and preterm cases without PPROM (Chao1, P=0.097; Shannon, P=0.66). **(B**) Principal coordinate analysis (PCoA) plot created at the ASV level generated by Weighted Unifrac distance, where the

values in parentheses show the percentages of total community variation explained ($P_{\text{Unweighted UniFrac}}$=0.47). The red dots represent preterm women with PPROM and the blue dots represent preterm women without PPROM.

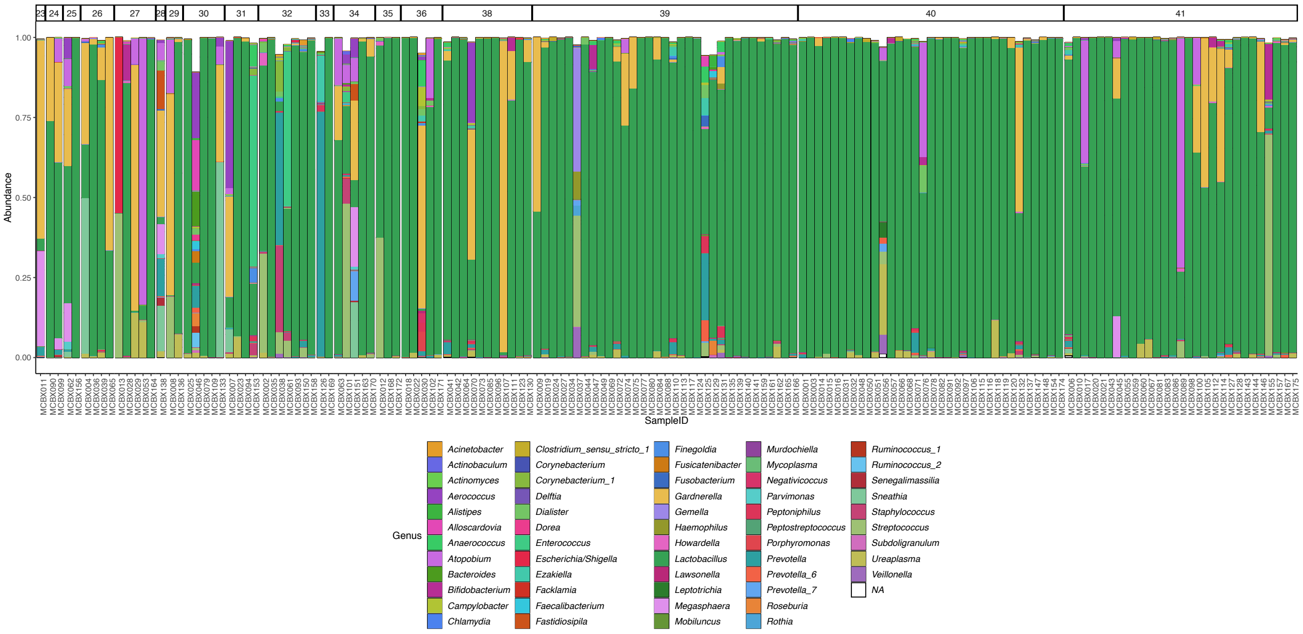

Supplementary Figure S4. Each bar represents a participant and each colored box a genus. The height of a color box represents the relative abundance of that genus within the sample according to a week of delivery. Lower abundance taxa are not shown.

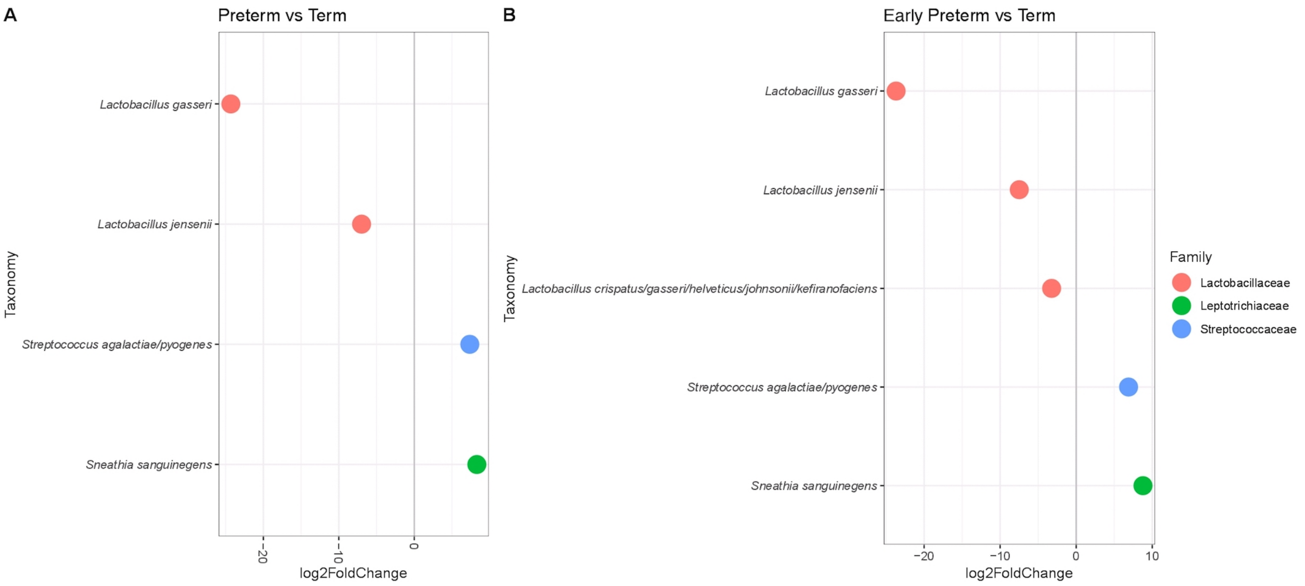

Supplementary Figure S5. Comparison of the differential abundance of vaginal microbial ASVs between different gestational age of delivery cohorts **(A)** Differential abundance: all preterm vs term. **(B)** Differential abundance: early preterm vs term. Each point represents an ASV. Only ASVs with significant differences ($P<0.05$, FDR-corrected) in log2 fold change are shown. ASVs with prevalence greater than 3% and >50 reads were included in the DESeq2 differential abundance analysis. *Lactobacillus crispatus/gasseri/helveticus/johnsonii/kefiranofaciens*: 100% *L. gasseri* according to BLAST.
